# Letter from the Editor in Chief

**DOI:** 10.19102/icrm.2024.15096

**Published:** 2024-09-15

**Authors:** Devi Nair


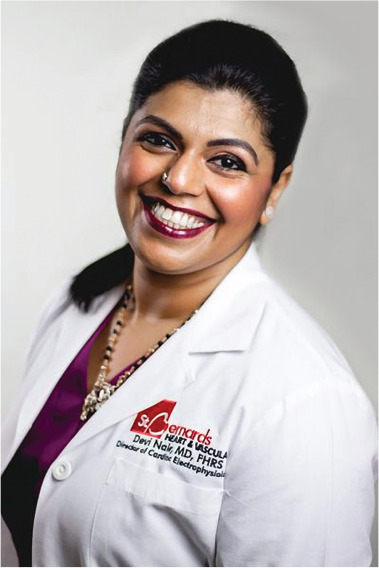


Dear readers,

I am excited to present the September 2024 issue of *The Journal of Innovations in Cardiac Rhythm Management*, where we explore groundbreaking advances in cardiovascular care. This edition showcases a rich variety of original research, reviews, and case studies, reflecting our commitment to advancing cardiac rhythm management through novel therapies and rigorous clinical inquiry.

We begin with a significant original research article, “Permanent Pacing Reduces Blood Pressure in Older Patients with Drug-resistant Hypertension: A New Pacing Paradigm?,” by Nguyen et al.^1^ This large retrospective study evaluated the impact of dual-chamber permanent pacing on patients with drug-resistant hypertension. The findings reveal that pacing not only improves both systolic and diastolic blood pressure but also reduces medication dependence. These results could pave the way for novel pacing strategies to better manage hypertensive patients who are resistant to pharmacotherapy.

Next, we turn to the comprehensive umbrella review by Jaya et al., “Efficacy and Safety of Intravenous Diltiazem Versus Metoprolol in the Management of Atrial Fibrillation with Rapid Ventricular Response in the Emergency Department.”^2^ This analysis highlights the superiority of intravenous diltiazem over intravenous metoprolol in rate control, despite an increased risk of hypotension. The review provides essential insights that may influence emergency department treatment protocols for atrial fibrillation patients with rapid ventricular responses, emphasizing the importance of personalized medicine.

Furthermore, this issue includes a cutting-edge case report by Gupta et al., “Simultaneous Isolation of Persistent Left Superior Vena Cava and Right Superior Vena Cava Using Pulsed-field Ablation with a Pentaspline Catheter for Recurrent Persistent Atrial Fibrillation.”^3^ This article illustrates the first simultaneous isolation of both vena cavae using pulsed-field ablation, a technology that minimizes damage to surrounding tissues and shows promise for complex atrial fibrillation ablation cases. The successful application of pulsed-field ablation in this case highlights the increasing role of non-thermal techniques in treating non–pulmonary vein triggers in atrial fibrillation management.

In addition to these impactful studies, this issue contains further innovative research and case studies that delve into the expanding frontiers of cardiac rhythm management. I am confident that the articles presented in this issue will inspire clinicians and researchers alike to pursue new methods and technologies to improve patient outcomes.

Looking ahead, in next month’s issue, I will provide an exclusive update on the late-breaking trials and studies presented at the 2024 Asia-Pacific Heart Rhythm Society conference in Sydney. Stay tuned as we bring you the latest in cutting-edge research from the forefront of cardiac electrophysiology.

As always, I would like to thank our dedicated authors, peer reviewers, and editorial team for their contributions to this month’s journal. We continue to strive for excellence in disseminating critical knowledge in cardiac rhythm management, and we look forward to sharing more exciting developments in the months to come.

Sincerely,


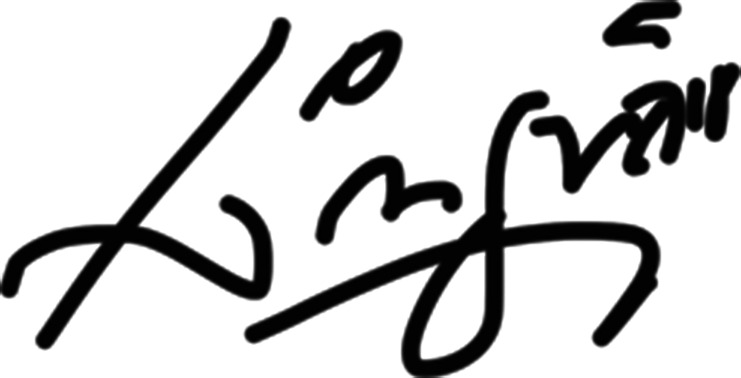


Dr. Devi Nair, md, facc, fhrs

Editor-in-Chief

*The Journal of Innovations in Cardiac Rhythm Management*

Director of the Cardiac Electrophysiology & Research,

St. Bernard’s Heart & Vascular Center, Jonesboro, AR, USA

White River Medical Center, Batesville, AR, USA

President/CEO, Arrhythmia Research Group

Clinical Adjunct Professor, University of Arkansas for Medical Sciences

Governor, Arkansas Chapter of American College of Cardiology

drdgnair@gmail.com
